# A Novel Loop Closure Detection Approach Using Simplified Structure for Low-Cost LiDAR

**DOI:** 10.3390/s20082299

**Published:** 2020-04-17

**Authors:** Qin Ye, Pengcheng Shi, Kunyuan Xu, Popo Gui, Shaoming Zhang

**Affiliations:** 1 College of Surveying and Geo-Informatics, Tongji University, Shanghai 200092, China; yeqin@tongji.edu.cn (Q.Y.); shipengchenglink@163.com (P.S.); 2 Zhongzhen Tonglu (Jiangsu) Robot Co., Ltd., Shanghai 201804, China; kyxu360@163.com (K.X.); guipopo@zztonglu.com (P.G.)

**Keywords:** simplified structure, point cloud registration suitability, hierarchical structure matching, loop closure detection, indoor scene

## Abstract

Reducing the cumulative error is a crucial task in simultaneous localization and mapping (SLAM). Usually, Loop Closure Detection (LCD) is exploited to accomplish this work for SLAM and robot navigation. With a fast and accurate loop detection, it can significantly improve global localization stability and reduce mapping errors. However, the LCD task based on point cloud still has some problems, such as over-reliance on high-resolution sensors, and poor detection efficiency and accuracy. Therefore, in this paper, we propose a novel and fast global LCD method using a low-cost 16 beam Lidar based on “Simplified Structure”. Firstly, we extract the “Simplified Structure” from the indoor point cloud, classify them into two levels, and manage the “Simplified Structure” hierarchically according to its structure salience. The “Simplified Structure” has simple feature geometry and can be exploited to capture the indoor stable structures. Secondly, we analyze the point cloud registration suitability with a pre-match, and present a hierarchical matching strategy with multiple geometric constraints in Euclidean Space to match two scans. Finally, we construct a multi-state loop evaluation model for a multi-level structure to determine whether the two candidate scans are a loop. In fact, our method also provides a transformation for point cloud registration with “Simplified Structure” when a loop is detected successfully. Experiments are carried out on three types of indoor environment. A 16 beam Lidar is used to collect data. The experimental results demonstrate that our method can detect global loop closures efficiently and accurately. The average global LCD precision, accuracy and negative are approximately 0.90, 0.96, and 0.97, respectively.

## 1. Introduction

Simultaneous Localization and Mapping (SLAM) with low-cost Light Detection and Ranging (Lidar) plays an important role in autonomous driving, artificial intelligence and virtual reality. With the development of robot technology, SLAM has attracted more and more attention and made some achievements [1,2,3,4]. For SLAM technology, various systems or platforms have been introduced, such as the Lidar system [5], stereo camera [6] and RGBD-camera [7]. Some technologies based on SLAM can contribute to the improvement of mapping accuracy, such as a Pseudo-GNSS/INS module integrated framework with probabilistic SLAM [8], a 2D SLAM system using low-cost Kinect Sensor [9], prediction-based SLAM (P-SLAM) [10], graph-based hierarchical SLAM framework [11], semi-direct visual-inertial SLAM framework [12], and a CPU-only pipeline for SLAM [13]. Similar to traditional data fusion technology [14], SLAM with data fusion technologies has also been developed accordingly, such as a fusion of the RGB image and Lidar point cloud [15,16,17]. A stereo visual inertial Lidar (VIL) SLAM incorporates tightly coupled stereo visual inertial odometry (VIO) with Lidar mapping and Lidar-enhanced visual loop closure [18]. Visual stereo image and 2D light detection and ranging (Lidar) data are incorporated into SLAM [19].

However, improving the robustness of SLAM is still a difficult task, especially in large indoor spaces, so the Pseudo-GNSS/INS module may not work well indoors. In fact, robot pose estimation is an iterative solution process using past scans. Without other auxiliary equipment, measurement errors will inevitably accumulate. LCD judges whether the robot returns to the place it has already passed. It is crucial to reduce the accumulated errors from odometry for long-running robots. After LCD, we can establish data association with past scans, then update the map. Here, we consider LCD as a matching task; loop closure may occur only if the scene structures are similar.

To date, there has been some research on LCD, which are generally divided into two categories according to data type: (1) LCD based on image data—visual sensor, formatted image with color information, which uses 2D features in images to express a 3D scene and then matches images based on the feature. However, the method is subject to illumination change. A well-known method is bag of words (BOW) [20], and LCD based on image data is performed [21,22,23]; (2) LCD based on point cloud data—laser sensor, discrete 3D points with accurate distance. Usually, registration work is done here, such as ICP [24] and GICP [25], then loops are detected with the registration results. Some other LCD methods combining images and point cloud are proposed, and visual CNN features are combined with submaps [26]. A key frame-based localization module is integrated into a particle filter-based position-tracking algorithm [27]. Compared with the first category, the second type of method is not subject to illumination change and has a distinct spatial topology, and viewpoint change is the only key factor. Its weakness is the massive data memory, and information loss for some specific structures.

In this paper, we propose an indoor global LCD method with a low-cost 16 beam Lidar. Firstly, we extract “simplified structure” in point cloud. Here, the “Simplified Structure” has simple geometry and are robust to describe the structured scene. It is superior to traditional feature description in extraction efficiency and robustness, even for low-cost Liar data. Then, we carry out a hierarchical matching based on “Simplified Structure” with multiple geometric constraints in Euclidean Space. Finally, global candidate loops are detected using a multi-state loop evaluation model of multi-level structure. Our main contributions are three-fold: (1) the “Simplified Structure” designed to capture robust structure in indoor point cloud has compact geometry and high extraction efficiency, and it offers a novel feature extraction for structured indoor scene; (2) The quantitative statistics of “Simplified Structure” can be exploited as a pre-judgment indicator for point cloud registering suitability, that is, to judge whether the point cloud can be registered or not. With the statistics, many non-loop scans are discarded, which significantly improves the efficiency and accuracy of global LCD; (3) our LCD method is valid and robust for the low-cost Lidar (16 lines) in an indoor environment. It overcomes the over-dependence on expensive high-resolution Lidar in previous methods.

The rest of the paper is organized as follows: Section 2 reviews related work. Section 3 introduces our global LCD method. Section 4 shows the experiment details. Section 5 discusses and analyzes our experiment. Finally, Section 6 concludes our work.

## 2. Related Works

Some LCD researches based on point cloud are generally divided into four categories:

(A) Non-feature description

LCD is merely performed with part of the original point cloud and no feature description is computed in this category. The closest points to Lidar in the point cloud are matched with improved Smith Waterman [28]. Point clouds without feature description are registered with ICP [29] and GICP [30] for LCD, and then registration results serve as major indicators for loops;

(B) Feature description

This category is classified into two parts according to the way of feature description. One is feature descriptor, the other is feature histogram. (1) Feature descriptor: descriptors, as the distinctive label for the point cloud, are used to match for LCD, and then a loop is detected when two descriptors are matched well. The major difference in each method lies in the descriptors and the matching approach for the descriptors. Researchers propose an improved FALKO interest point and GLAROT descriptor, then they analyze three feature association algorithms methods: Random Sample Consensus (Ransac), Correspondence Graph, and Hough Data Association [31]. On the point cloud range image, a local distance map describing the neighborhood structure is used as a descriptor for LOG interest points [32]. Researchers project a 3D point cloud to multiple 2D planes and generate a density signature for points for each of the planes, then use the left and right singular vectors of these signatures as the descriptor [33]. (2) Feature histogram: most histograms originate from some statistical attributes on point cloud, and the loops are detected based on the histogram similarity. Many researchers have proposed various histograms under different applications. A projection function assigns an additional attribute value for points, and then a normalized histogram generated from the point density in each projection function value interval [34]. Space is divided into many cells that are labeled with line, plane, sphere, etc. Point number in each cell category constitutes the histogram, then the multi-scale one is formed at a different distance [35]. Researchers construct the histograms similarly to [35] and determine the candidate loop scan based on the Normal Distributions Transform (NDT) registration [36]. A histogram that discretizes the dot products of normal and vertical direction into 101 bins for every point and counts quantity in each interval is designed [37]. Generally, the state of histograms is related to the density of point cloud to some extent. Some research about the data distribution characteristics of Lidar under different densities are published [38,39,40,41,42,43];

(C) Deep learning

The random forest classifier determines whether the matched point cloud represents the whole or part of the same object with a predefined feature space [44,45]. Synchronous adversarial feature learning with a dual Bi-GAN that associates the 2D Bi-GAN with 3D Bi-GAN is proposed, and it can learn abstract attributes from different dimensions without any label data [46]. A semi-manual representation learning method based on a Siamese convolution neural network is proposed, and it manages LCD as a similarity modeling problem [47]. With multiple point cloud features, AdaBoost is used to detect candidate loop scans, which are further screened in back-end optimization [48];

(D) Other methods

The pose map is constructed with the odometry data, and then neighborhood path geometry is matched to determine loops [49]. Using rasterized map matching, LCD is carried out between raster maps using branch-and-bound [50]. The indoor corridor is divided into four states and the longest common subsequence matching and hu-moment-based contour match are adopted [49]. Researchers detect loop closures based on a grid map representation of the environment, and the map is created via Rao-Blackwellized particle filtering [51].

However, some issues for LCD still exist in an indoor environment: (1) LCD based on a visual scheme is seriously affected by illumination change; (2) LCD with multi-beam Lidar (64) suffers from mass data memory and calculation, and since the information of a low-cost 16 beam Lidar is less than that of multi-beam Lidar, the previous LCD methods are inapplicable, and little LCD research is performed on 16 beam Lidar; (3) pre-analyzing the registering suitability of point cloud scans plays an important role for global LCD, so discarding those scans with low registering suitability will improve the efficiency and accuracy of LCD, however, there is not much research on registering the suitability of a point cloud.

Our method is applicable to 16 beam Lidar and not affected by illumination change. It overcomes the dependence on high-resolution point cloud sensors. This makes it possible for SLAM to generate desirable results with those low-cost sensors. The method can capture an indoor robust structure with less computation complexity. Through quantitative analysis of the structure in the scene, we obtain the statistics of the extracted “Simplified Structure” and can remove a large number of non-loop scans in global LCD. It is extremely applicable to some structured indoor scenes that have a robust structure, such as walls or cylinder pillars. In the process of LCD, our method can determine the loop through the scan-to-scan match, and there is no need to exploit other additional point cloud maps.

## 3. Methodology

**Figure 1:**
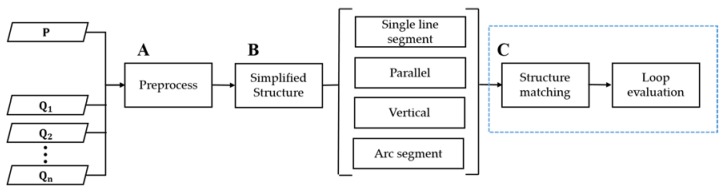
Flow chart. P is the current point cloud scan input. $Q_{1}Q_{2}\ldots Q_{n}$ are all candidate point cloud scans saved locally. A B and C are three main stages-Preprocessing, Simplified Structure extraction and Loop evaluation respectively.

Our LCD main flowchart is shown in Figure 1. There are three main stages:

(A) Preprocessing: the raw point cloud is processed to remove some interference objects, such as ceiling and ground. Then, the point cloud is corrected in the Z direction according to the point cloud distribution. Finally, the point cloud is orthographically projected on the XOY plane;
(B) Simplified Structure extraction: the scene structures are classified according to their structure salience. In addition, we adopt different extraction methods in different types of Simplified Structure;
(C) Loop evaluation: a pre-match is performed to remove the candidate scans whose structure number varies greatly. Then, we utilize a loop evaluation module to analyze the matching state of the scans to detect loop closure.

### 3.1. Simplified Structure

There are two difficulties in point cloud feature extraction: (1) large computation; (2) unrobust feature extraction. Some researchers exploit normals to capture plane structure in data [52,53], which is not enough for indoor environment because it lacks a quantitative description for structural distribution. To overcome this shortcoming, we propose a “Simplified Structure” to capture the stable structure in point cloud and exploit some salient attributes to describe the “Simplified Structure” distribution, such as the length of the wall and the radius of the cylinder. We obtained some new structure attributes when the structure became complicated, for example, the distance between two walls is also a robust description for the corridor beside the length of the walls. Some specific structural attributes are discussed below.

**Figure 2:**
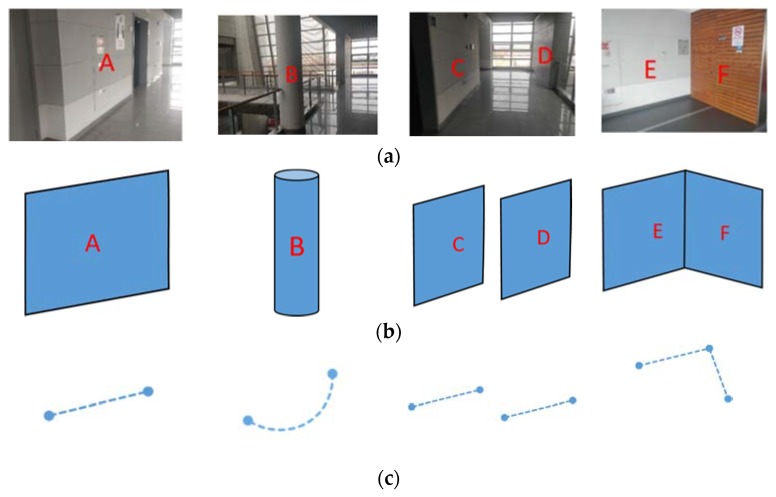
Simplified structure in indoor environment. (**a**) the rgb images of real scene; (**b**) stable structure; (**c**) Simplified Structure result. Column 1: Single line; Column 2: Arc segment; Column 3: Parallel; Column 4: Vertical.

We classified “Simplified Structure” into two levels. The first level, Single Line Segment (expressed as Single Line in the following), is also a basic unit for some other structures. On the second level is the line segment pairs composed of two single lines, such as parallel and vertical, and the Arc segment. Figure 2 shows the simplified results for a common structure in an indoor scene. It has to be mentioned that some glass structures may exist in some indoor scenes, which may impair the data accuracy. However, the proposed “Simplified Structure” can capture a robust wall structure which is subjected little to the glass. If a large glass structure and insufficient “Simplified Structure” exist, these point cloud scans will not be qualified to become a candidate loop scan in LCD, because we pay more attention to accuracy than recall. However, if the glass structure dominates in the scene, the performance of our LCD may be limited.

**Figure 3:**
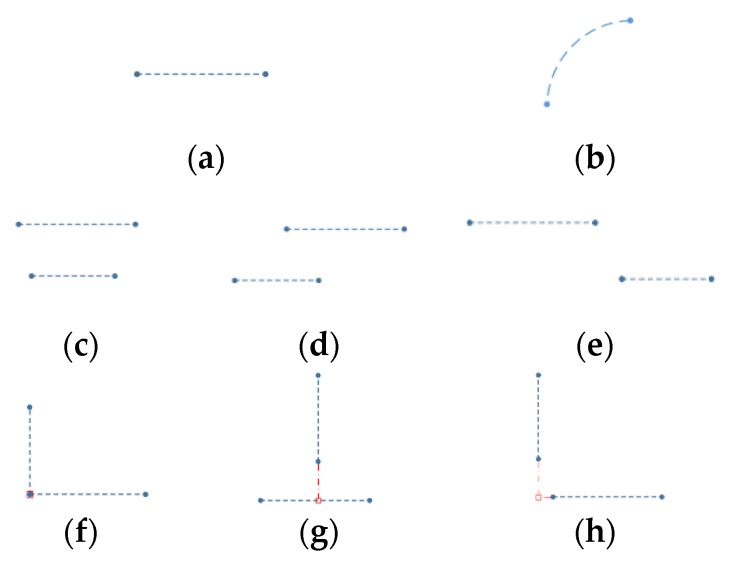
Categories of Simplified Structure. (**a**) Single line. (**b**) Arc segment; (**c**) Complete overlap parallel; (**d**) Partial overlap parallel; (**e**) Non-overlap Parallel; (**f**) Complete real vertical; (**g**) Partial real vertical; (**h**) Virtual vertical.

In Figure 3, the “Simplified Structure” is further classified according to the geometric and positional relationship of a structure unit. (a) shows the 1st level, and (b)–(h) the 2nd level.

(1) Single Line (Figure 3a): The simplest structural attributes—two vertices, line length, line parameters. This refers to the wall structure; vertices and length are its range distribution description, and line parameters for its direction description;

(2) Arc Segment (Figure 3b): This stands for the cylindrical structure’s attributes—the arc center, radius, chord length, and geometric points of the arc, as well as a midpoint on the arc and the two endpoints farthest from Lidar on both side of the midpoint;

(3) Parallel (Figure 3c–e): Approximate parallel line pairs are classified into three types—complete overlap, partial overlap and non-overlap—according to the overlap relationship of two lines. Attributes—two single lines (first-level structure), two line overlap ratios, and overlap type;

(4) Vertical (Figure 3f–h): Approximate vertical line pairs are also classified into three types—complete real vertical, partial real vertical and virtual vertical—according to the position of perpendicular foot and two lines. Attributes—two single lines, a perpendicular foot and vertical type.

### 3.2. Preprocessing

The preprocessing prepares for LCD, including pass-through filtering, *Z*-axis correction and orthographic projection. After the preprocessing, the projection point cloud on XOY plane is obtained. The operating environment of Lidar can be considered approximately horizontal.

#### 3.2.1. Pass-through Filtering

Pass-through filtering filter out points by two height thresholds, then we get $P_{z-\mathrm{filter}}\;\left(N_{z-filter}\right)$ in Formula (1). $N_{z-filter}$ is the number of points in point cloud $P_{z-\mathrm{filter}}$; similar expression is used below. Ground and ceiling points are preliminarily removed here.

(1) $$P_{z-\mathrm{filter}}=\{p\;|H_{min}<p.z<H_{max},\;p\in \;P_{z-correct}\}$$

where $H_{min}$ and $H_{max}$ are two height threshold sets based on the height of the experiment platform.

#### 3.2.2. *Z*-Axis Correction

Although the operating environment of Lidar is approximately horizontal, some deviations still exist. *Z*-axis correction is used to eliminate the deviation. Covariance matrix (COV) is constructed in Formulas (2) and (3). Then, two principal directions of the point cloud are obtained through Singular Value Decomposition (SVD) on COV. They are aligned with the horizontal plane to obtain the corrected point cloud $P_{z-correct}$

(2) $$P_{z-filter}^{center}=\frac{1}{N_{z-filter}}\underset{i=1,2\;\ldots \;N_{z-filter}\;}{\sum }P_{z-filter}^{i}$$

(3) $$COV=\underset{i=1,2\;\ldots \;N_{z-filter}\;}{\sum }{\left(P_{z-filter}^{i}-P_{z-filter}^{center}\right)}^{T}*(P_{z-filter}^{i}-P_{z-filter}^{center})$$

where $P_{z-filter}^{i}$ is the point cloud after pass-through filtering; $N_{z-filter}$ is the point number of $P_{z-filter}^{i}$; $P_{z-filter}^{center}$ is the point cloud center in $P_{z-filter}^{i}$.

#### 3.2.3. Orthographic Projection

**Figure 4:**
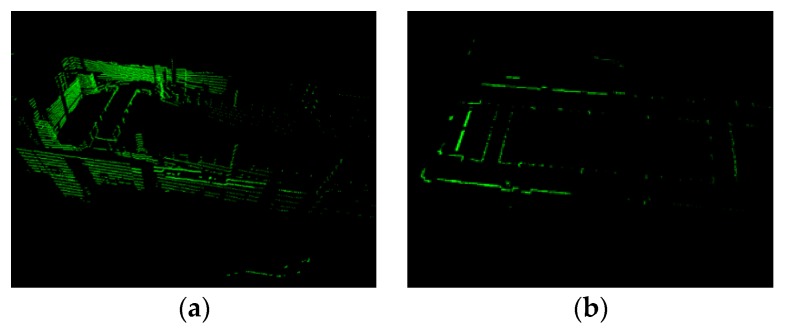
Preprocess result (**a**) original point cloud (**b**) preprocessing result. The scan is sampled from dataset 1 (Section 4).

Using Formula (4), $P_{z-correct}$ is orthographically projected onto the XOY plane. Then, we obtain the projected point cloud $P_{project}\;$(Figure 4b)

(4) $$P_{project}=\{p\;|\;p.x=q.x,\;\;p.y=q.y,\;\;p.z=0,\;\;q\in \;P_{z-correct}\}$$

where *p* and *q* represent points in $P_{project}$ and $P_{z-correct}$, respectively.

### 3.3. Hierarchical Extraction of Scene Simplified Structure

#### 3.3.1. First-Level Structure

**Figure 5:**
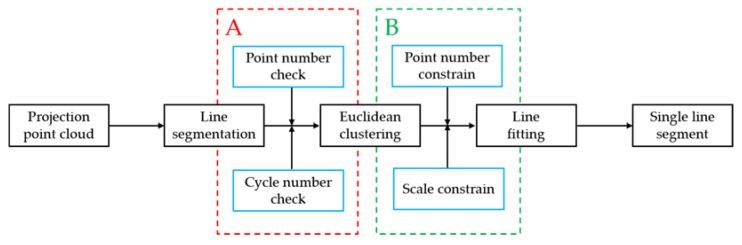
Flow chart of the first-level structure extraction. A is the module—“cycle self-checking”, and B is the module—“quantitative analysis and reconstruction of units”.

Figure 5 is the flowchart of the first-level structure extraction. We propose a cycle segmentation strategy with two modules—A: cycle self-checking; B: quantitative analysis and reconstruction of units. In each segmentation, the largest point cloud subset that satisfies the same line distribution is extracted in the current remaining point cloud. The cycle self-checking module prevents the whole process from falling into an indefinite cycle. The quantitative analysis and reconstruction module ensure the robustness of a structure through removing trivial structures. The reason we adopted this strategy is that linear-distributed wall structures are major components in an indoor environment, and piecewise walls are consistent in the direction based on architectural structure form.

(1) Line segmentation

In Formula (5), $Line_{pre}$ is extracted through Ransac [54], and $P_{left}$ is updated after each successful line segmentation. Line parameters are computed. The cycle self-checking module includes two parts: (a) point number check, where $N_{ls}$(minimum points) is set to judge whether the line extraction is complete. Most line segment structures in the scene have already been extracted when $Line_{pre}$ is small enough; (b) cycle number check: $C_{iter\_max}$ (maximum cycle number) prevents the process from falling into an infinite cycle

(5) $$P_{left}=\{p\;|\;p\in P_{project},\;p\notin \;Line_{pre}\}$$

where $P_{left}$ is the remaining point cloud in $P_{project}$ after each successful line segmentation; $Line_{pre}\;$, the largest point cloud subset that meets the same line distribution in $P_{left}$, and $P_{left}$ is just $P_{project}$ in the first line segmentation;

(2) Euclidean Clustering

**Figure 6:**
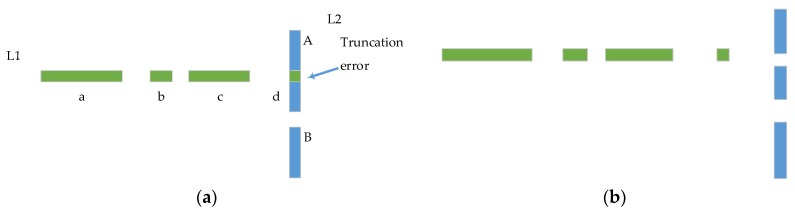
Truncation error. (**a**) Structure before truncation error; (**b**) Structure after truncation error; L1 and L2 are two $Line_{pre}$ in Section 3.3.1 (1), a b c and d are the Euclidean clustering results of L1 in Section 3.3.1 (1), and A B for the L2. Blue line segment L2-A is blocked by L1 because of the truncation error.

Each $Line_{pre}$ is classified into multiple $P_{line\_cluster}^{i}$(*i* = 1, 2…K) by Euclidean Clustering. The quantitative analysis and reconstruction of unit includes two parts: (a) point number constrain—to some extent, point numbers indicate its validity, so $N_{pr}$ is set. If the point number of $P_{line\_cluster}^{i}$ is less than $N_{pr}$, it means that this $P_{line\_cluster}^{i}$ is meaningless to scene representation, the $P_{line\_cluster}^{i}$ object is a small scattered structure, or the structure is broken by truncation error. Figure 6 show the truncation error breaks’ stable structure; (b) Scale constrain—$L_{pr}$ is set to examine the diagonal length of the minimum bounding rectangle of $P_{line\_cluster}^{i}$. After quantitative analysis and the reconstruction of the unit, some $P_{line\_cluster}^{i}$ will be put back into $P_{left}$ for the next line segmentation.

(3) Line fitting

**Figure 7:**
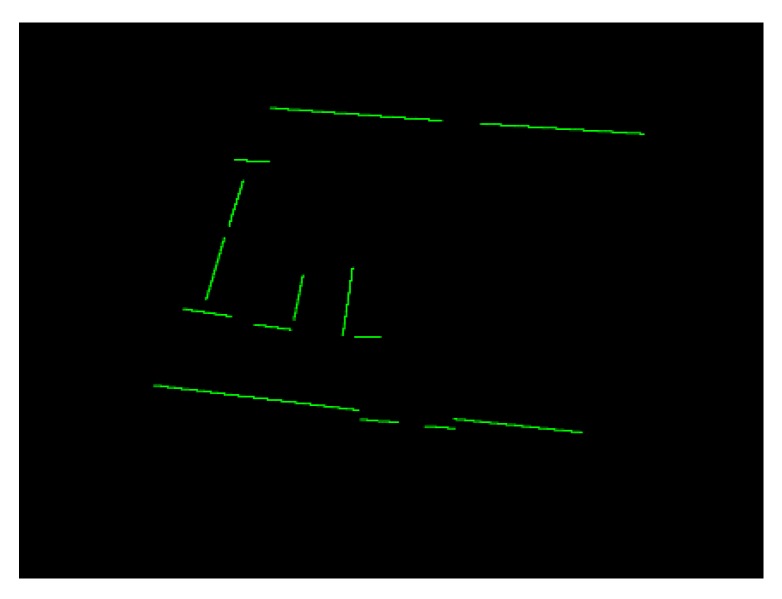
First-level structure—single line.

We obtain line segment vertices in $P_{line\_cluster}^{i}$ through Ransac. We proposed a “Boundary Projection Fitting Algorithm” (Algorithm 1) to improve fitting accuracy. In Algorithm 1, P is $P_{line\_cluster}^{i}$, *resolution* is distance interpolated resolution in line segment, and the output is single line (first-level “Simplified Structure”). The algorithm performs projection analysis on $P_{line\_cluster}^{i}$, and the result is shown in Figure 7.
Alogorithm 1 Boundary projection in line fitting$1:\;\mathrm{Input}\;P={\left\{p_{i}\in R^{n}\;\right\}}_{1:N}\;,resolution$2: Output Single Line $3:\;Line_{parameter}\left\{a,\;b,\;c\right\},\;\;Line\leftarrow \mathrm{Ransac}\;\mathrm{Line}\;\mathrm{fitting}\left(P\right)$ $4:\;idx_{min}\leftarrow 0,idx_{max}\leftarrow 0$5: if line gradient>1$6:\;for\;P_{i}\;\in \;P\;do$$7:\;Project_{i}\leftarrow \mathrm{project}\;P_{i}\;\mathrm{to}\;Line$8: end for $9:\;idx_{min}\leftarrow \mathrm{idx}\;\mathrm{of}\;Y_{min}\;,\;idx_{max}\leftarrow \mathrm{idx}\;\mathrm{of}\;Y_{max},\;\mathrm{in}\;Project\;$$10:\;N_{point}\;\leftarrow \;(Project_{idx_{max}}.x-Project_{idx_{min}}.x)\;/\;resolution$11: end if12: else $13:\;for\;P_{i}\;\in \;P\;do\;$$14:\;Project_{i}\leftarrow \mathrm{project}\;P_{i}\;\mathrm{to}\;Line$15: end for $16:\;idx_{min}\leftarrow \mathrm{idx}\;\mathrm{of}\;X_{min},\;idx_{max}\leftarrow \mathrm{idx}\;\mathrm{of}\;X_{max},\;\;\mathrm{in}\;Project$$17:\;N_{point}\;\leftarrow \;(Project_{idx_{max}}.y-Project_{idx_{min}}.y)\;/\;resolution$18: end else$19:\;line\_endpoint1\leftarrow Project_{idx_{min}}$, $line\_endpoint2\leftarrow Project_{idx_{max}}$20: Single Line ←interpolate point according to line_endpoint1, line_endpoint2, N

#### 3.3.2. Second-Level Structure (Parallel, Vertical)

The structure pairs are more stable than the first-level structure in the feature description. Some single line pairs are exploited as the second-level structure (Parallel, Vertical).

(1) In Formula (6), all possible single line pairs are reserved as

(6) $$\left\{(Singleline_{i},Singleline_{j})\;|i\neq j,i,j\in N_{Singleline}\right\}$$

where $N_{Singleline}$ is the number of the first-level structure;

(2) In Formula (7), angle classification, two types of structure pairs (*Parallel Vertical*) are selected based on the angle between two single lines (Cos). We set $Cos_{p}$ to 0.866 and $Cos_{v}$ to 0.259.

(7) $$\{\begin{array}{c} Parallel\\ Vertical \end{array}\begin{array}{c} \;\;\;Cos>Cos_{p}\;\\ \;\;Cos<Cos_{v} \end{array}$$

where $Cos_{p}$ and $Cos_{v}$ are the cosine value thresholds for Parallel and Vertical, respectively;

(3) Attributes statistics: **Parallel**—(a) Distance: average distance of two vertices to the other single line. (b) Overlapping types: we compute the ratio of overlapping length in each single line length, and classified overlapping types into three types—complete overlapping, partial overlapping and non-overlapping. If one single line is far away from the other, the pair will be discarded. (c) The attributes that two single line have. **Vertical**—(a) perpendicular foot. (b) Vertical types: three types—point outside the two, point in one but outside the other and point in two according to the position of the perpendicular foot and two single lines. (c) The attributes that two first-level structures had.

#### 3.3.3. Second-Level Structure (Arc segment)

**Figure 8:**
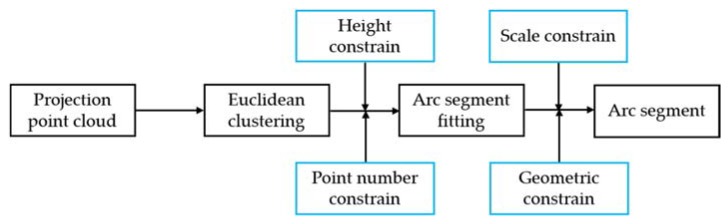
Flow chart of the second-level structure—arc segment extraction.

In arc segment extraction, we adopted a serial process with several quantitative analysis, as shown in Figure 8.

(1) Euclidean Clustering

$P_{project}$ is classified into clusters $P_{arc-cluster}^{i}\left(i=1,2\ldots N_{arc-cluster}\right)$ by Euclidean clustering. Then, we introduced two quantitative analysis constrain: (a) height constrain—some $P_{arc-cluster}^{i}$ clusters, such as pedestrian and potted plants, are discarded with height threshold $H_{cluster}$. An index correspondence mechanism is presented here to obtain the z coordinate as Formula (8); (b) Point number constrain—some trivial clusters are discarded by point numbers threshold $N_{ac}$

(8) $$\left\{j⟷k\;|index:\;j\in \;P_{arc-cluster},\;k\in \;P_{z-filter}\;\;\right\}\;$$

where $P_{arc-cluster}$ is a point cloud cluster after Euclidean clustering; $P_{z-filter}$ is the point cloud after pass-through filtering in Section 3.2.1; j, k are point index in $P_{arc-cluster}$, $P_{z-filter}$.

(2) Arc segment Fitting

**Figure 9:**
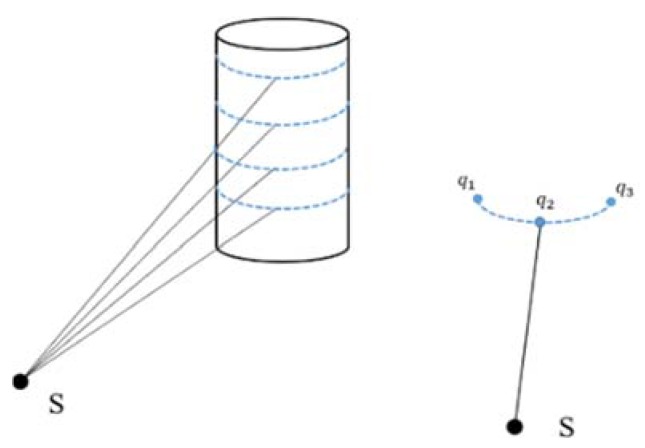
Relative Position of Lidar and Cylindrical Structure. S represents the Lidar; *q*_1_ and *q*_3_ are two arc endpoints, *q*_2_ is the arc midpoint.

Ransac is exploited again to fit the arc in each $P_{arc-cluster}^{i}$. (a) Scale constrain: some linear clusters are removed with maximum radius threshold $R_{max},$ because line segment is a special arc structure with a large radius. (b) Geometric constrain: three points (one arc midpoint and two arc endpoints) are exploited to verify the arc characteristics. In Figure 9, we define a complete arc that should have an arc midpoint ($q_{2}$) and two arc endpoints ($q_{1}q_{3}$). $q_{2}$ is the closest point to Lidar, and $q_{1}$ and $q_{3}$ are the two farthest points on each side of $q_{2}$; they resemble an isosceles triangle. Incomplete arc segments caused by occlusion or other reasons will be discarded here.

### 3.4. Loop Closure Detection

Compared to traditional feature extraction methods, “Simplified Structure” indicates the distribution characteristics of the surrounding environment in a simpler way. Furthermore, the number and type of structure is also illustrated. With the number and types of “Simplified Structure”, we can approximate the position state, which will significantly improve the performance of global LCD.

**Figure 10:**
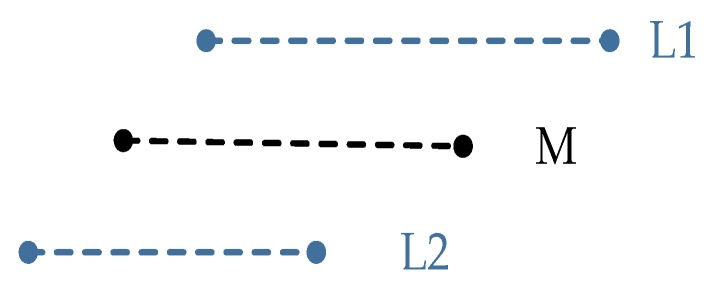
Midline in Parallel. L1 and L2 are a Parallel structure; M is the midline.

Inspired by Smith Waterman [55] and Hungarian Algorithm [56], we present a hierarchical matching strategy with multiple similarity metrics in Euclidean space, and the matching rate of “Simplified Structure” indicates scene similarity. The similarity metrics include geometric metric, topological metric, and matching metric. The geometric metric is the attribute similarity measure for each type of structure. Topological metric is the primary relative position similarity measure between matching pairs. Matching metric is the precise relative position similarity measure between matching pairs after transformation. First, candidate-matching pairs satisfying geometric similarity are selected. Secondly, topological analysis is performed under the same “Simplified Structure”. Finally, the error equations on transformation parameters are constructed, and the similarity state for the same type of “Simplified Structure” is evaluated. Some auxiliary feature attributes can be utilized here, for example, in Figure 10, we calculate a midline to assist matching analysis in the parallel structure.

#### 3.4.1. Pre-Match

After Section 3.3, we have extracted all “Simplified Structures” in each scan. Then, we roughly determine the registration suitability of two scans through a quantitative analysis of the Simplified Structure. Since the “Simplified Structure” can capture the stable scene structure, if the number of structures differs greatly, then the two scans cannot be registered, and they must not be a loop. Pre-match quantitative analysis includes two parts: (a) the existence of structure—if a certain type of structure exists in one scene but not in the other, then they must not be a loop; (b) quantitative difference in structure—the more the structure quantity differs, the lower the probability of loop is. Many non-loop scan pairs are discarded here, and much time is saved because time-consuming structure matching is not performed. As a crucial advantage of our Simplified Structure, we can eliminate many non-loop scans just by quantitative analysis in this step.

Due to the robustness of the “Simplified Structure”, this Pre-match is extremely suitable to analyze the point cloud registration suitability. It exploits the quantitative analysis of the structure to determine the similarity between point cloud scans roughly instead of through traditional overlapping analysis. It utilizes the intuitive Simplified Structure number to determine whether the registration can be carried out successfully. This provides an excellent pre-analysis for point cloud registration.

#### 3.4.2. Structure Hierarchical Matching

**Figure 11:**
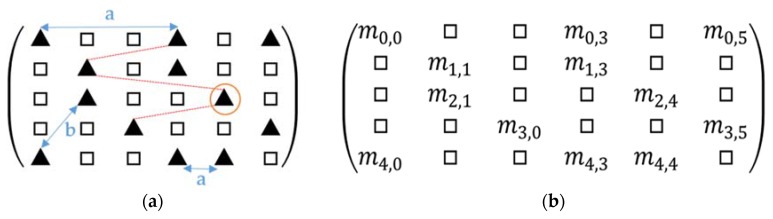
Matching analysis matrix. (**a**) Candidate match matrix (Candidate); (**b**) Matching number matrix *(*Match ). Row and column in two matrixes represent the index of Simplified Structure in source and target data, respectively. Solid triangle represents a candidate match structure pair, whereas hollow square represents a non-match structure pair; red dashed line—topological analysis of match pair; blue solid line a—the two structure pair that cannot exist in the same match queue; blue solid line b, the two structure pair can exist in the same match queue; $m_{i,j}$, the maximum match pair number found in Candidate for ${\mathrm{Candidate}}_{i,j}$.

Firstly, for each type of “Simplified Structure” in two scans, we construct a candidate similarity matrix (Candidate) according to structure attributes. Its row and col represent a structure index for two scans (as shown in Figure 11a). Secondly, we find the longest matching queue for each matching pair (solid triangle in Figure 11a) in Candidate. Thirdly, a matching number matrix (Match*)* is constructed (Figure 11b), where $m_{i,j}$ means the maximum matching pair number in Candidate for structure matching pair $Candidate_{i,j}$. The maximum value of Match is the maximum matching number for this type of structure.

Four parts need to be explained in the longest matching queue search process. (1) In the process for $Candidate_{i,j}$, which is the first pair in the matching queue, and we search all possible pairs in Candidate. $M_{i,j}$ is the maximum pair number of the queue for $Candidate_{i,j}$ (Figure 11b). (2) The structure pairs in the same row or col cannot exist in one matching queue, for example, the blue solid line a in Figure 11a, $Candidate_{0,0}\;Candidate_{0,3}$, cannot exist in the same matching queue because the structure pair is one-to-one. (3) Topological metric is first considered here, which can ensure that the matching queue satisfies the relative position relation. Topological metrics include point-to-line distance, point-to-point distance, and angle between line and line. (4) Error evaluation: in Formulas (9) and (10), point-to-point distance and point-to-line distance of structure matching pairs are used

(9) $$cost\;funtion1:\;\underset{T}{arcmin}\sum \frac{A*T\left(x\right)+B*T\left(y\right)+C}{\sqrt{A^{2}+B^{2}}}$$

(10) $$cost\;function2:\underset{T}{arcmin}\sqrt{{\left(X-T\left(x\right)\right)}^{2}+{\left(Y-T\left(y\right)\right)}^{2}}$$

where *A*, *B*, and *C* are the parameters of line equation; *x* and *y* are the coordinates in the source scan; *X* and *Y* are the coordinates in the target scan. T(x) and T(y) are the coordinates of *x* and *y* after transformation. T is the transformation parameter, and it can also be used for the point cloud registration.

#### 3.4.3. Loop Evaluation

**Figure 12:**
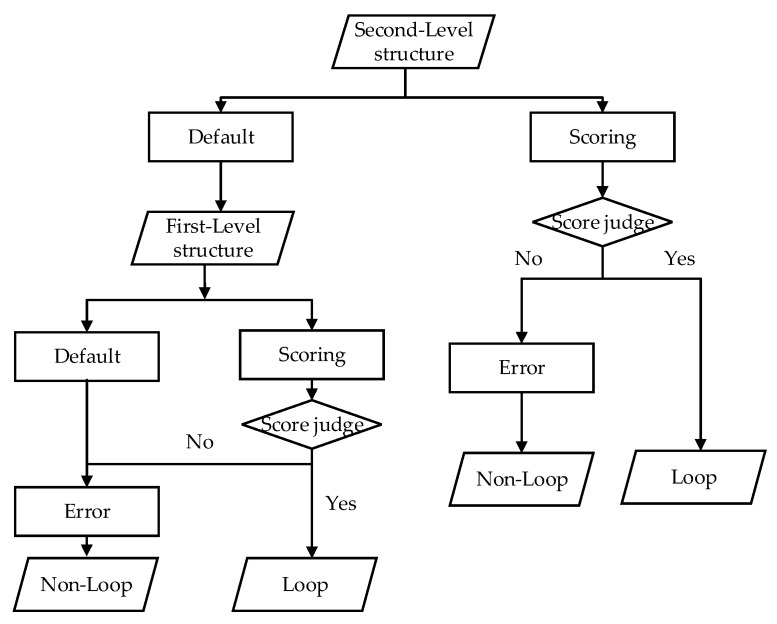
Flow chart of loop evaluation. “Score judge” is to judge whether score is higher than the score threshold ($S_{sim}$ ) in scoring state. Default, Scoring and Error are three evaluation states.

A multi-state loop evaluation model of multi-level structure is constructed in Figure 12. It scores each type of structure. There are three evaluation states for each type of Simplified Structure: (1) default state—structure quantity is insufficient (parallel-2, vertical-1, arc segment-2, single line-5); (2) scoring state—score is computed in Formula (11) to see if both two scans have sufficient structure and some structure pairs are matched well; (3) error state—a special state, which takes place in two situations, one where the score is less than $S_{sim}$ in the scoring state for Simplified Structure, the other where a default state occurs in the first-level structure. If an error state occurs in one type of Simplified Structure, the two scans will be regarded as non-loop immediately. Before evaluation, the evaluation states of all Simplified Structures are initialized as default state. As shown in Figure 12, the second-level structure is evaluated first, and if all of them remain in default state after evaluation, the first-level structure will be evaluated. The corresponding structure in two scans will be considered as similar if their score is higher than $S_{sim}$ in scoring state; we set $S_{sim}$ to 60%. On the contrary, the evaluation state of two scans are set to error if the score is less than $S_{sim}$. When the error state occurs, the current candidate scan will be discarded and the next scan matching will start.

(11) $$score=\frac{N_{m}}{N_{q}}\times 100\%\;$$

where $N_{m}$ is the maximum matching number of the simplified structure in two data; $N_{q}$ is the maximum number of the Simplified Structure in two data.

If two scans are a loop closure, their score should be higher than $S_{sim}$ for the Simplified Structure in scoring state. For global LCD, most scans are non-loop and it will be extremely time-consuming if we perform structure matching. In our method, massive non-loop scans can be eliminated through pre-match. The multi-state loop evaluation model improves the accuracy and efficiency of LCD through the hierarchical management of structure. Our LCD method depends on the Simplified Structure; it can detect loop scans efficiently through a quantitative analysis of the structure. It is extremely effective in a scene that has some robust structure.

In our LCD, if two-point cloud scans are identified as loop after loop evaluation, then we can also acquire the transformation parameters based on the cost function in Formulas (9) and (10), and we adopt the least-square solution.

## 4. Experiment

### 4.1. Experimental Platform and Data Description

**Figure 13:**
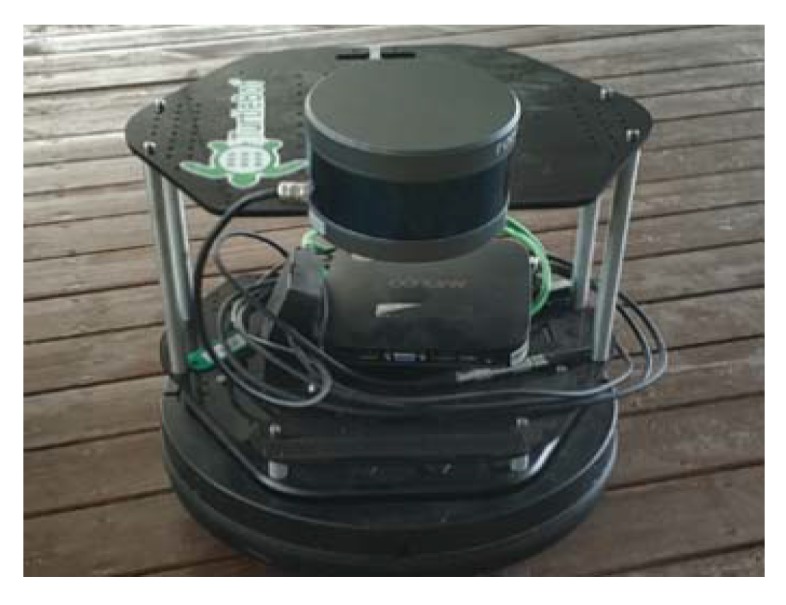
16 beam Lidar in our experiment. A 16 beam Lidar is set on the Turtlebot platform.

Our global LCD experiment was carried out offline; the 16 beam Lidar and platform in the experiment is shown in Figure 13. Differences between our platform and Pseudo GNSS/INS setup are three-fold: (1) applicable scene—pseudo GNSS/INS is not suitable for long-time localization in large indoor scenes (although the accuracy is higher for outdoor). Our experiment setup works well under bad (or even no) lighting conditions; (2) platform cost—our main experimental platform composes of a 16 beam Lidar and has no other equipment, and the cost is less than GNSS/INS setup; (3) external signal—we don’t need any external signals to perform localization, and satellite signal is required in Pseudo GNSS/INS setup.

**Figure 14:**
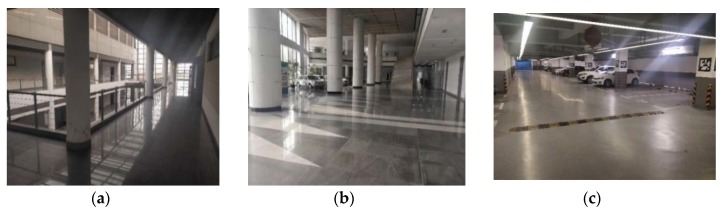
(**a**) Dataset 1—corridor; (**b**) Dataset 2—hall; (**c**) Dataset 3—underground parking lot.

**Figure 15:**
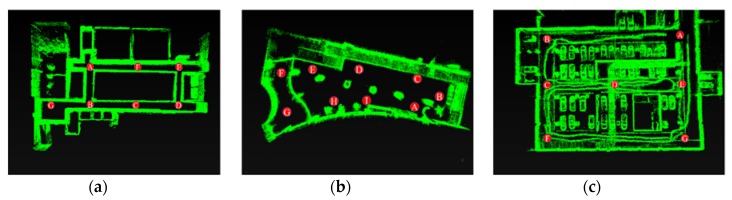
Lidar trajectory. (**a**) Dataset 1, A-B-D-E-A-B-G-B-D-E-A; (**b**) Dataset 2, A-B-C-D-E-F-G-H-I-A-I-H-E-D; (**c**) Dataset 3, A-B-C-D-E-D-C-F-G- A.

In Figure 14, there were three typical indoor scenes in our experiment and their Lidar trajectories were shown in Figure 15. Dataset 1 was a common indoor corridor scene; dataset 2 was an indoor hall; dataset 3 was an underground parking lot. We collected 4500 (dataset 1), 547 (dataset 2) and 10,400 (dataset 3) point cloud scans in three datasets, respectively. The trajectory in Dataset 1 was a round route; the trajectory in dataset 2 and dataset 3 included a back-and-forth route. We obtained the ground truth of each scan by Laser SLAM and obtained the relative pose to the first scan. A laptop with Intel Core i7-5500U CPU @2.40 GHz 2.39 GHz and 8.0 GB of RAM was applied in our experiment. The key scan was sampled to a lower computation. Here, the key scan interval is different in three datasets because of the different number of collected data scans. Dataset 1: a key scan every five scans, dataset 2: every scan is a key one, dataset 3: a key scan for every ten scans. If our method is applied to online SLAM, constant key scan interval could be exploited.

### 4.2. Parameter Setting

The experimental parameters (including some error thresholds) were classified by modules as Table 1, preprocess parameters, Simplified Structure parameters and loop detection parameters. Some critical parameters were introduced here: (a) preprocess—some specific parameters such as $H_{min}$ and $H_{max}$ needed to be adjusted according to the installation height of Lidar, and $H_{min}$ can be set to the approximate installation height; (b) Simplified Structure—$N_{pl}$ and $N_{iter}$, the termination conditions of the process, were empirically set to 300 and 50, respectively. Parameters here ensured the accuracy of the Simplified Structure and they were mainly for accuracy. It was verified that these parameters change little for different indoor environments; (c) loop evaluation—parameters here were mainly for the similarity measurements of the Simplified Structure. The higher the $S_{sim}$ was set, the more similar the two scenes were, and we set $S_{sim}\;to\;60\%$.

### 4.3. Experiment Results of Structure Extraction and Matching

Limited by the paper length, only two loop scans were sampled as analysis example in each dataset. In the experiment, the ground truth mentioned in Section 4.1 was exploited to verify our LCD method performance. In Table 2, we listed number of Simplified Structure, number of structure matching and time consumption for the example scans in three dataset. Time consumption of Simplified Structure extraction and matching was also computed.

#### 4.3.1. Dataset 1

**Figure 16:**
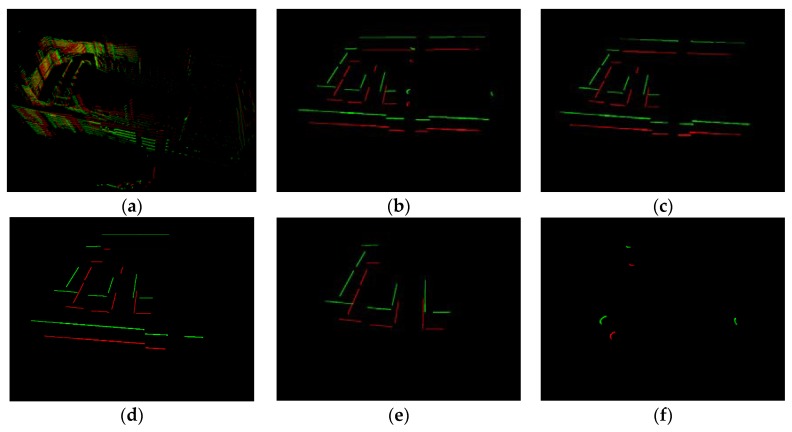
Matching results of structure in Dataset 1. (**a**) The 978th and 3618th scan original point cloud. (**b**) All Simplified Structure; (**c**) Single line; (**d**) Parallel; (**e**) Vertical; (**f**) Arc segment. Green—the 978th scan; Red—the 3618th scan.

Dataset 1 was a corridor scene; the 978th and 3618th scan were sampled. Figure 16 showed the original data (a) and Simplified Structure (b–f). For the visual effect of matching results, we aligned two scans and kept 20 cm in z direction. The two scans are around D in Lidar trajectory (Figure 15a). All kinds of Simplified Structure were detected here. The structures of the two scans were similar and matched well on the visible effect (Figure 16b–f). In Table 2, number of Simplified Structure in two scans were almost same and all matching ratios were higher than 60%, especially the vertical, which was 100%.

#### 4.3.2. Dataset 2

**Figure 17:**
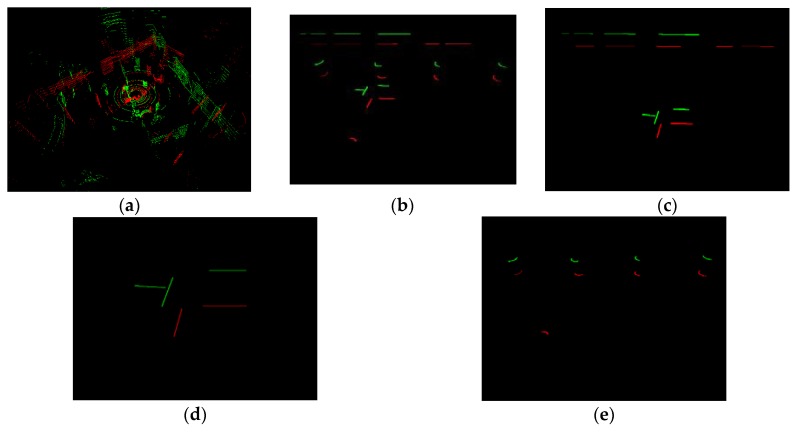
Structure matching results in Dataset 2. (**a**) The 419th and 13th scan. (**b**) All Simplified Structure; (**c**) Single line; (**d**) Vertical; (**e**) Arc segment. Green—the 419th scan; Red—the 13th scan.

Dataset 2 was a spacious open hall scene, and there were a few stable structures in some data scans. The 13th and 419th scan were sampled. In Figure 17, the Parallel and Vertical in two scans were lower than in dataset 1, and they were in default state after loop evaluation. Arc segments were extracted in two scans. In Figure 17, we can see that the sole vertical in the 13th scan was matched well with one vertical in the 419th scan. All arc segments in the two scans matched well. Single lines were also matched in order to upgrade the result reliability, and the same excellent matching result was shown in Table 2—seven pairs of single lines among two scans were well-matched.

#### 4.3.3. Dataset 3

**Figure 18:**
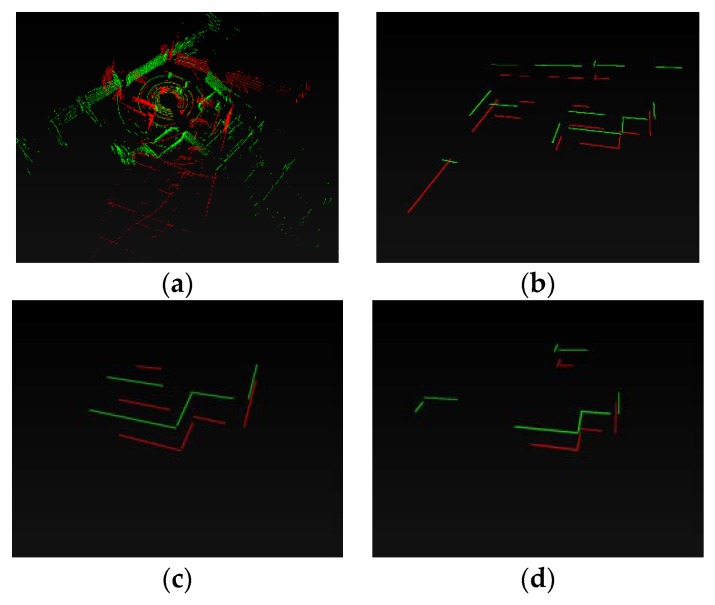
Matching results of structure in Dataset 3. (**a**) The 10150th and 490th scan original point cloud. (**b**) All Simplified Structures (Single line); (**c**) Parallel; (**d**) Vertical; Green—the 10150th scan; Red—the 490th scan. The robust arc segment was not detected, so all Simplified Structures were equivalent to a single line segment.

Dataset 3 was an undergrounding parking lot. It had two challenges, moving vehicles and pillars that partition parking space. The pillars may result in structure occlusion in some close position. The 10150th and 490th scan were sampled. The arc segment was in default state after loop evaluation. In Figure 18, four pairs of Vertical and three pairs of Parallel were matched. The matching similarity of the second level (Parallel and Vertical) reached over 70%; these two scans could be considered as a loop. Similar to Section 4.3.2, the first-level structure was matched to improve the reliability. In Table 2, there were 13 and 15 first-level structures in two scans, respectively, and 12 pairs were well-matched.

## 5. Discussion

### 5.1. Similarity Matrix

**Figure 19:**
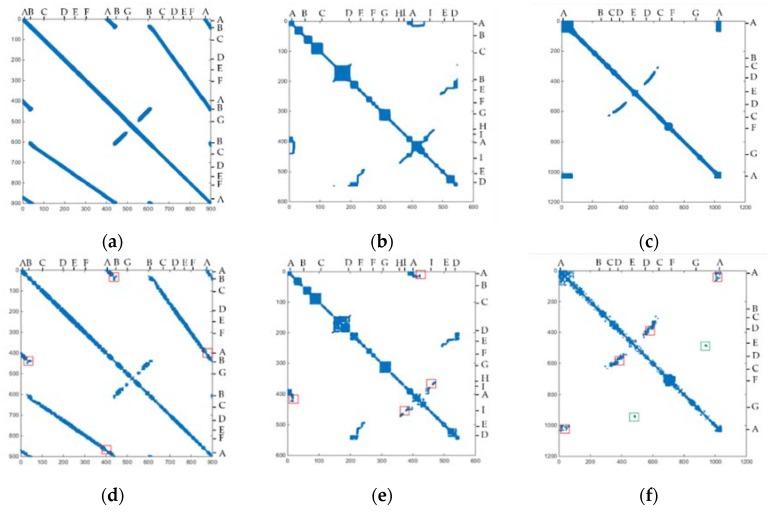
Similarity matrix. (**a**–**c**) Similarity matrix from ground truth; (**d**–**f**) similarity matrix from our method. Red rectangle represents undetected loop scans; Green rectangle represents wrong loop scans. Column 1: dataset 1; column 2: dataset 2; column 3: dataset 3. The row and column in matrix were key scan sequence number. The number represents the key scan serial number. This means that 1 represents the 5th scan point cloud in dataset 1, the first scan in dataset 2, and the 10th scan in dataset 3. A, B…I in the figure refers to the position in three datasets.

The similarity matrix [37,38] was also applied in our method evaluation; the colored area represented similar scans. In Figure 19, our similarity matrix is consistent with the ground truth similarity matrix. The number on the figure is the key scan serial number. Dataset 1 (Figure 19a,d): there were three loops near A and B, and two near the other parts, which was also detected in our method. G was an empty spacious vicinity near the elevator, deviating from the corridor, where the Lidar moved slowly for a period of time, resulting in many similar areas from the 400th to 500th scan. Dataset 2 (Figure 19b,e): the loop in A E H I was detected effectively in our experiment. Dataset 3 (Figure 19c,f): CDE was a back-and-forth route where the loop mainly occurred. They were also labeled in our similarity matrix. However, due to the occlusion of scene structure and other reasons, there were some defects in the contour of similar matrixes. In Figure 19, we marked some loops that were undetected in our method (red rectangle), such as AB in Dataset 1, AI in Dataset 2, AD in Dataset 3. In Dataset 3, the 900th and 500th key scan were mistaken as a loop due to the high similarity of parking space structure (green rectangle). There are only few false cases in our experiments. Since the purpose of our method was to find the global loop fast and accurately, missing loops did not impair the method performance as long as loop detection was accurate enough. These false loops can be further eliminated through comparative analysis among candidate loop scans.

### 5.2. Error Metrics

Our task was to ensure the accuracy of detected loops and eliminate any false loops as much as possible. Obviously, there would be many negative matches when global LCD was performed in many scans. That is why Negative Predictive Value (*NPV*) and Accuracy (*ACC*) were also adopted besides Precision (*PPV*) and Recall (*TPR*). PPV in Formula (12) refers to the rate of detecting true loop, TPR in Formula (13) indicated that the rate of all loops had been found, *NPV* in Formula (14) indicated the exclusion rate of a non-loop, and *ACC* in Formula (15) represented the rate of our right decision. *NPV* is an effective metric to verify the capability to exclude the non-loop accurately in global LCD. Our method was evaluated quantitatively with the four indicators

(12) $$PPV=\frac{TP}{TP+FP}$$

(13) $$TPR=\frac{TP}{TP+FN}$$

(14) $$NPV=\frac{TN}{TN+FN}$$

(15) $$ACC=\frac{TP+TN}{TP+TN+FP+FN}\;$$

where TP, true positive, is the correctly detected matching scan, FP, false positive, is the incorrectly detected matching scan, *TN*, true negative, is the correctly detected non-matching scan, FN, false negative, is the incorrectly detected non-matching scan.

**Figure 20:**
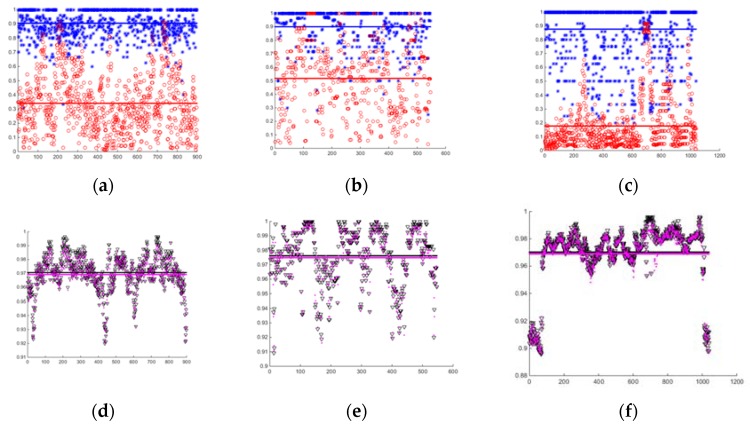
Error metrics of method. (**a**–**c**) are the PPV and TPR for dataset 1 2 3 respectively. (**d**–**f**) are the NPV and ACC for dataset 1 2 3 respectively. Column 1: dataset 1; column 2: dataset 2; column3: dataset3. Blue asterisk: PPV; red hollow circle: TPR; black hollow triangle: NPV; magenta dot: ACC.

In Table 3, both the *NPV* average and *ACC* average in three datasets reached over 0.95, the *PPV* average in dataset 1, 2 were higher than 0.90 and that in dataset 3 was 0.8761. Low *TPR* was the biggest drawback, but it would not affect the performance of LCD, as mentioned above. The precision of some scans was less than 0.5, as shown in Figure 20a–c, and there may be two possible reasons: (1) there are few Simplified Structures in those places, and (2) the laser was obstructed due to some interference factors such as pedestrians or moving vehicles; in Figure 20a–c, the low-precision scans are close to each other. The situation happened more, and some false loops were found in dataset 3.

## 6. Conclusions

In this paper, we propose an offline global LCD method for low-cost Lidar (16 lines) in indoor scene to improve the robustness and efficiency of indoor SLAM. Adopting the proposed “Simplified Structure” is effective to capture a robust point cloud structure. It utilizes a few points to transform environment information that is hard to distinguish visually into an intelligible geometric structure. It is extremely suitable for a structural indoor scene that has some piecewise walls, robust pillars or other robust structures. In our method, if two scans are identified successfully, we can also obtain the transformation parameters that can be used for point cloud registration. Our method can offer a desirable global LCD performance and the precision of our method basically satisfies the accuracy requirements in SLAM.

The “Simplified Structure” we propose is a robust feature description for structured indoor point cloud scene. Our proposed LCD method benefits from the “Simplified Structure”. If a Simplified Structure is applied in laser SLAM, the data memory will be reduced significantly. We test the method on three typical datasets and acquire desirable results. Our global LCD method is especially suitable for a sparse Lidar point cloud and detects global loop successfully. Its precision reaches nearly 0.9 despite the low recall. Although the experiment is performed offline, its accuracy and efficiency meet the localization performance requirements in SLAM. The registering suitability analysis based on a “Simplified Structure” is very valued for point cloud registration work.

Improving the instability of the positioning system is an important task in the field of robots. Our LCD method can effectively improve the robot localization performance to ensure the regular operation process according the error analysis. Generally, simplified structured information is abundant in an indoor environment, which is very beneficial to our method. Our method has several remarkable characteristics, low cost, fast detection, and no Pseudo-GNSS/INS module. This is very beneficial to some low-speed robots, and our fast LCD method can improve the positioning accuracy of the robot and avoid accidents. This method is also effective for low-cost Lidar, which can avoid being applied to expensive equipment and wasting resources. It can be applied to robot products integrating a slam framework.

The objects described by “Simplified Structure” are those stable scene structures. The structure extraction result is susceptible to moving objects, because they will break the structural integrity. Therefore, a dynamic interference objects removal technology needs to be studied. In addition, we will try to apply our global LCD method to an online SLAM system. We will consider more types of Simplified Structures and extend the method to some compact outdoor scenes.

## Figures and Tables

**Table 1:** Overview of parameters in experiment.

Module	Parameters	Value
Preprocess	Maximum height $H_{max}$	−0.2 m
	Minimum height $H_{min}$	2.5 m
Simplified Structure extraction	Minimum point number of segmented line $N_{ls}$	300
	Maximum cycle number $C_{iter\_max}$	50
	Minimum point number of units $N_{pr}$	50
	Minimum unit length $L_{pr}$	1 m
	Minimum arc cluster points $N_{ac}$	50
	Maximum Arc radius $R_{max}$	3 m
	Maximum line segmentation error $L_{pl}$	0.1 m
	Maximum Arc segment fitting error $L_{arc}$	0.1 m
	Maximum line fitting error $L_{lf}$	0.03 m
Loop evaluation	Maximum Geometric similarity length error $L_{gs}$	0.2 m
	Maximum topology error $L_{ta}$	0.2 m
	Maximum cumulative matching error $L_{ae}$	0.2 m
	Structure similar score $S_{sim}$	60%
	Maximum distance of Loop $L_{tr}$	10 m

**Table 2:** Quantization Results and Matching Status of Simplified Structure and time cost for example data.

		Simplified Structure				Matching Number				Time	
Dataset	Scan Number	A	V	P	S	A	V	P	S	Structure Extraction/s	Structure Matching/s
1	978	3	4	8	14	2	4	7	−0.01	0.901875	0.046108
	3618	2	4	10	15	66.7%	100%	70%	-	0.638146	
2	419	4	2	0	7	4	−0.01	−0.01	7	0.684369	0.04199
	13	5	1	0	7	80%	-	-	100%	0.49625	
3	10,150	--	5	3	13	-0.01	4	3	12	1.23388	0.023985
	490	--	4	4	15	-	80%	75%	80%	0.985223	

A result of −0.01 means that the Simplified Structure is in default state in loop evaluation because it has an insufficient structure for matching analysis. A, V, P and S were Arc segment, Vertical, Parallel and single line, respectively. The structure matching time here only referred to the time consumption of the sampled two scans. The percentage (XX%) is the score for the structure matching.

**Table 3:** The average of the four error metrics.

Dataset	Error Metric Average			
	Precision (*PPV*)	Recall (*TPR*)	Negative Predictive Value (*NPV*)	Accuracy (*ACC*)
Dataset 1	0.9050	0.3405	0.9706	0.9692
Dataset 2	0.9002	0.5177	0.9762	0.9748
Dataset 3	0.8761	0.1774	0.9702	0.9690
